# Passing Events and Topics

**Published:** 1921-10

**Authors:** 


### PASSING EVENTS AND TOPICS.

A memorial is being raised to Miss Lucy Robinson, one of the founders of the Incorporated Society of Trained Masseuses, who, it will be remembered, died last February. Not only was she a founder of this Soceity, which has now become the Chartered Society of Massage and Medical Gymnastics, but she is recognised to have been an important motive-force in its progress and development, and she continued her untiring work on its behalf to the end of her life. Her work for midwives is also well known to all who have followed the progress of the movement to improve the standard of midwifery and the status of midwives. The memorial will take the form of three bursaries to assist towards the training of students in massage and remedial gymnastics. It is hoped to raise £1,000, and to award the first bursary for training in September 1923. Subscriptions will be received by the honorary secretaries, Miss Field, 2 Albany Terrace, N.W. 1, and Miss Mary Lindsay, 17 Cromwell Road, S.W.

---

Presentations to matrons on their retirement are quite a usual thing, but a presentation that is now in contemplation to Miss Deane, who after thirty-five years' service is retiring from the matronship of the East Suffolk and Ipswich Hospital, is of peculiar interest. Several presentations have already been made to her from the hospital staff and others, but a desire has now been expressed that the general public in the neighbourhood should have an opportunity to show their esteem and gratitude to Miss Deane by a personal gift. Services such as those of Miss Deane are too often taken for granted, and the intention to express in a tangible form the appreciation of Ipswich and the neighbourhood does credit to the reputation of the district which the East Suffolk Hospital serves.

---

The Rev. H. A. Birks, late Vicar of Kingsbridge and Chairman of the Kingsbridge Cottage Hospital, on leaving the town and retiring from the Chairmanship has made a very generous gift to the town. He has presented to it the Cottage Hospital for the use of the sick and the poor of the district in memory of his mother. At the recent annual meeting Mr. Birks explained that in the deed of gift he had stipulated that the hospital's constitution must not be changed for seven years, except by a vote of two-thirds of the subscribers. At the end of that time, the Committee had discretion, if it seemed desirable to secure larger premises, to sell the present property for the benefit of the funds. Should the hospital fail from any cause, the property would pass to the Vicar and churchwardens of St. Edmund's Church for the benefit of the social work connected therewith; but he hoped that the hospital would be carried on. A vote of thanks to the Chairman for his splendid gift was passed, and good wishes were expressed for his happiness in his retirement.

---

The death last month of Sir Ernest Cassel removed a wealthy benefactor who will be remembered for more than the amount of his gifts. Though these were proportionate to his income, and therefore large, his capacity was not limited to the signing of a cheque. Unlike the run of millionaires, he had sane ideas, and did not limit himself to supporting established institutions. He wished to found new ones for new purposes, and to supply needs neglected in his time. The sanatorium established by him at Midhurst and his recent foundation of a hospital for nervous disorders are examples whose original aim, we hope, will not be lost upon other wealthy persons desiring to be thought philanthropists. Sir Ernest was one of those City magnates honoured by the notice of King Edward, whose shrewd eye probably detected in the financier a man of mind as well as money. Sir Ernest took the best advice within his reach; and, since the art of spending money is one which the self-made millionaire has rarely the time or the capacity to study, he deserves to be ranked with the philanthropists of the nineteenth century, a band of earnest men to whom the voluntary system in all its branches owes much.

---

Despite its visitors, Bournemouth has found difficulty in supporting its hospitals, and recent schemes include one for organising collections from the businesses and trades of the place. The present list of supporters is incomplete, and it is believed that many suppose the scheme to be directed only to those who employ a numerous staff. Of course, that is a misconception. It is the small business, the one-man staff, that is also desired to contribute. The scheme entitles the staff to hospital benefits, and the one man is as much in need of them as any group of employees. In London the Hospital Saturday Fund has collections in businesses where as few as three, we believe, are employed. If three is not too few in London, one is enough in any country town.

---

The new Braintree and Bocking Cottage Hospital, which is the gift of Mr. W. J. Courtauld, is virtually finished, in spite of the unavoidable delays experienced since the foundation stone was laid fifteen months ago. It contains twelve beds for accidents and surgical cases, and its equipment includes an $x$-ray installation, and even a motor-shed. The new hospital succeeds the "old" institution at Bocking, which the present donor's father presented to the district. An endowment fund has been started, and, since the new hospital was formed to meet the needs of a growing industrial population, it is much to be hoped that a system of weekly contributions will be started. Without that help the maintenance cannot be secure.

**PASSING EVENTS AND TOPICS—(*cont.*).**

The annual conference of the Chartered Society of Massage and Medical Gymnastics, which took place on October 6, 7, and 8, was a particularly successful one, the meetings being well attended and the addresses and demonstrations closely followed. That they deserved to be will be realised by a recital of the names of those who so generously gave of their time to deliver lectures. They were Sir James Purves Stewart, who spoke on "Disseminated Sclerosis"; Miss Angove, who gave demonstrations at Guy's Hospital on "The Treatment of Empyema" and "The Tests and Progression of Muscular Work in Heart Cases"; Dr. A. F. Hurst, on "Massage and Exercises in Gastro-Intestinal Disorders"; Sir Henry J. Gauvain on "The Treatment of Surgical Tuberculosis"; Miss Stansfeld, of the Bedford Physical Training College, who gave a lecture-demonstration on "The Importance of Physical Fitness for the Professional Woman"; Dr. J. B. Mennell, on "Passive Movement"; and Dr. Crichton Miller, on "The Physiological Significance of Rest." These lectures, we understand, will all be published in successive issues of the *Journal* of the Chartered Society.

---

Mr. Edward Goodwin, a working man of Derby, has been presented with a gold medal for his successful efforts on behalf of the Derbyshire Hospital for Sick Children. Since June 1909 he has collected £503 odd for the hospital, in recognition of which the working-men's committee has made this presentation.

---

The war did much to bring home to the public some measure of realisation of the nature of the work that is being carried on daily within the walls of our great voluntary hospitals. But the ignorance that still exists is great, and where there is ignorance the attempt to arouse sympathy which shall express itself in financial support cannot fail to be ineffective. The Middlesex Hospital is, therefore, to be congratulated on the plan which its authorities are now quietly putting into practice with the view of bringing the hospital into direct touch with the residents and workers in its immediate neighbourhood, and thus spreading a knowledge of its work, not only in the treatment of patients, but in the advancement of medical education and science. They have instituted a series of evening conversaziones, to which the employees of the trading firms in the district are invited, when various departments of the hospital are opened for the inspection of the guests and various processes explained by those in charge. The first of these gatherings on a large scale was held this month. Some three hundred persons were present, and displayed a keen interest in all they saw and heard, which included continuous demonstrations in the *x*-ray and bacteriological departments and short talks on "Radium" by Professor W. S. Lazarus-Barlow. An organ recital was given in the hospital chapel, and refreshments and music were provided in the students' common room. We shall be glad to hear of other hospitals where similar efforts at the enlightenment of the public are being made. It is not enough to throw a hospital open for inspection on the occasion of a fete, or a prize distribution, or some other function. The people to be interested, to be enlightened, are the workers, and these can only come in the evenings, and will not come unless they are specially invited.

---

Dr. Charles Porter, the medical officer of health of St. Marylebone, has been waging war against rats, and in his annual report for 1920 he writes: The rat has been said to be the most expensive animal that man maintains, and, unlike most vermin, has no redeeming feature. As a food consumer and destructive agent he is easily first. Unfortunately, in proportion to his size, he has the largest brain of all the mammals, and, after centuries of oppression, he is out to survive. This makes the problem more difficult, and it is necessary to vary the methods employed to further his destruction.

---

During the final stages of the war an extremely simple and portable form of disinfecting apparatus was designed by Colonel Lelean, Professor of Hygiene of the Royal Army Medical College, London, and was actually used with front-line troops for the destruction of vermin and their ova in clothing. In its simplest form the apparatus consists of a steam-tight sack which is filled with infected clothing, blankets, etc., and the mouth then partially closed by a draw-string.

This sack is suspended from any convenient point in an inverted position, connection being made by hose from the top of the sack to the boiler, and steam is admitted at slightly over atmospheric pressure. As the steam enters at the top of the sack the air in the sack is gradually expelled from the sack mouth at bottom until the whole of the sack and its contents are saturated with live steam, which liberates its latent heat by condensation and sterilises the contents; 2.05 tons of textiles, or 1,000 Army blankets can be completely and thoroughly sterilised in eight hours working on a consumption of $5\frac{1}{2}$ gallons of oil; the boiler can, however, be placed on a kitchen fire or other handy means of heating.

The apparatus is being made in various forms by Messrs. Meldrums, Ltd., Timperley, near Manchester; the simplest portable form consists of a small boiler, without fittings or gauges of any kind, heated by means of an oil lamp; a steam-tight sack suspended by means of a cord. The boiler is provided with a vertical filling pipe which admits the water near the bottom of the boiler and so acts as a safety-valve and low-water alarm; a branch at the top of the boiler is connected up to the top of the sack by a flexible hose, and after steam has been raised the sack is filled until the steam finds its way out at the bottom of the sack. The sack may be packed tight with articles, or, in case of the disinfection of suits of clothing, etc., a circular frame is provided which fits inside the sack and on which hooks are provided on which to hang the garments. The process of disinfection takes only a few minutes, and the clothes when removed are only slightly damp but so hot that they require only a very short time to dry thoroughly.

The plant is also made in a permanent form, with boiler, a collapsible canvas chamber, and an adjustable roof, or in the form of a horse-drawn portable plant. Fully descriptive and priced lists can be had on application to the manufacturers.

